# Management of A Case of Endobronchial Blood Clot in the Post Operative Period

**Published:** 2009-04

**Authors:** Jyotirmoy Das, Saurabh Mahajan, Mukesh Kumar Samplay, Naresh Kumar Aggarwal

**Affiliations:** 1 Clinical Associate, Department of Anaesthesia, Fortis Flt. Lt. Rajan Dhall Hospital, Vasant Kunj, New Delhi; 2 Clinical Associate, Department of Anaesthesia, Fortis Flt. Lt. Rajan Dhall Hospital, Vasant Kunj, New Delhi; 3 Associate Consultant, Department of Anaesthesia, Fortis Flt. Lt. Rajan Dhall Hospital, Vasant Kunj, New Delhi; 4 Consultant, Department of Anaesthesia, Fortis Flt. Lt. Rajan Dhall Hospital, Vasant Kunj, New Delhi

**Keywords:** Endobronchial blood clot, Pulmonary collapse, Bronchoscopy

## Abstract

**Summary:**

Endobronchial blood clot is an unusual cause of airway obstruction leading to lung collapse in the postoperative period. It is not always easy to pin point the exact etiology in the presence of multiple risk factors. Pulmonary collapse can herald the onset of severe haemodynamic derangements and hypoxemia. So, early identification and management is crucial for preventing catastrophic complications. Various modalities have been described in the literature for removing the obstructing clot and re-expansion of the lung.

We present a case of postoperative left lung collapse by an obstructing endobronchial blood clot in a patient undergoing coronary artery bypass graft surgery.

## Introduction

Respiratory tract obstruction due to blood clot may result in life-threatening airway obstruction or pulmonary collapse. In 1929, Wilson first reported a case of endobronchial obstruction from blood clot resulting in lobar collapse^1^. Pasteur reported sixteen cases of postoperative massive atelectasis and stated that this comprised about eight percent of the overall postoperative complications.

Persistent pulmonary collapse following an episode of airway bleed should raise the suspicion of an obstructing blood clot^2^. If not diagnosed early, gross haemodynamic instability and desaturation may follow. A high degree of suspicion is the key in such a clinical presentation, especially when a recent history of hemoptysis is present. Management in the form of bronchoscopic evacuation of the obstructing clot helps in re-expansion of the affected lung segments and prevent further complications.

## Case report

**Fig 1:**
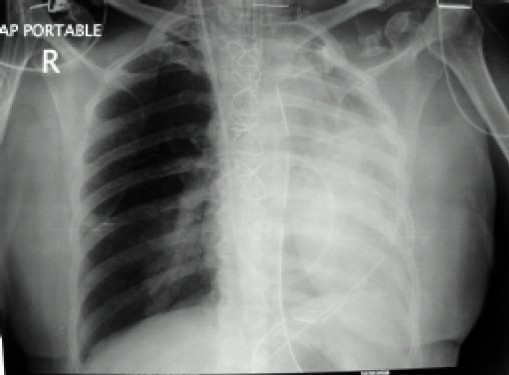
Chest radiograph showing homogenous opacity in left lung field with mediastinal shift towards the left side suggesting left-sided lung collapse.

**Fig 2:**
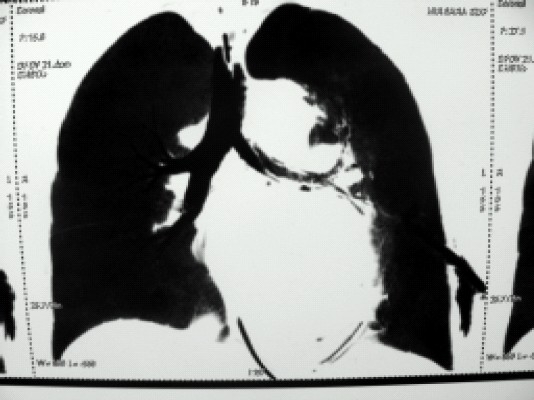
Computed tomography of the chest showing a clot in the left main bronchus arising from trachea (tip of ETT).

**Fig 3:**
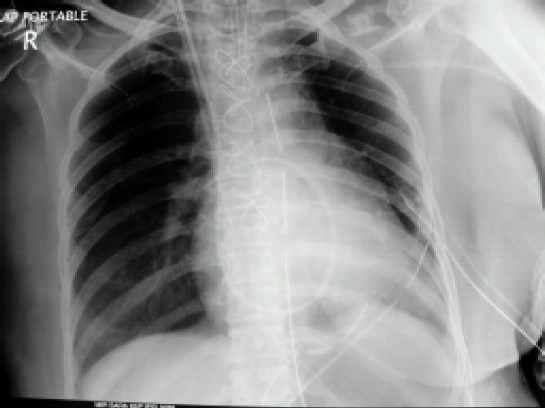
Chest radiograph showing a well-expanded lung after bronchoscopic clot evacuation.

A 62-year-old female weighing 65 kg underwent coronary artery bypass graft surgery following myocardial infarction Non ST Elevation Myocardial Infarction (NSTEMI) one month back and recurrent chest pain. She had history of hypertension for two years and diabetes mellitus type II for ten years with associated neuropathy, nephropathy and retinopathy. She was on oral metoprolol, atorvastatin, isosorbid mononitrate, aspirin, clopidogrel and subcutaneous insulin therapy. There was no history of tuberculosis, chronic obstructive airway disease or haemoptysis. Preliminary blood investigations showed haemoglobin 11.2 g/dl, serum creatinine 2.4 mg/dl, prothrombin time 18.2 seconds, international normalized ratio 1.48 with a normal bleeding time and clotting time. Chest radiograph was normal. Two-dimensional echocardiography revealed a left ventricular ejection fraction of 40 per cent and triple vessel disease was evident in coronary angiography. Post angiography, tab aspirin and clopidogrel was stopped and enoxaparin sodium 40 mg subcutaneous injection was started. Patient was scheduled for coronary artery bypass surgery after three days. Enoxaparin was stopped 24 hours prior to surgery. In the operation theatre, baseline blood gas analysis at an inspired oxygen fraction (FiO2) of 0.6 showed a pH 7.36, PCO2 36.3 mmHg, PO2 78.7 mmHg, oxygen saturation 95.1 per cent with a base deficit of 3.8. After induction with incremental doses of fentanyl (total dose of 500 mcg), midazolam 1 mg, and rocuronium (60 mg), trachea was intubated with 7.5 mm internal diameter endotracheal tube (ETT) at the first attempt. Airway pressure was 20 mmHg with a positive end expiratory pressure (PEEP) of 5 mmHg. After induction of anaesthesia, a triple lumen central venous catheter (7.5F) and thermo dilution Swan Ganz catheter (7.5F) was inserted via the right internal jugular vein. Baseline pulmonary artery pressure was 38/ 21 mmHg with a wedge pressure of 20 mmHg. Coronary artery grafting was done using left internal mammary artery and saphenous vein as conduits. Chest tubes were placed and adequate haemostasis achieved with activated clotting time of 145 seconds. During the process of sternal closure, it was noticed that airway pressures were gradually rising despite the patient being adequately paralyzed and no visible kink in the ETT. Pulse oximetry reading (SpO2) gradually dropped to 86% and airway pressure recorded a peak of 42 mmHg. At this point, blood was seen in the ETT lumen. Gentle ETT suction was done to remove approximately 10 ml of blood while oral cavity was found to be clear. The ETT refilled with blood, although the amount was less than before. This was also sucked out. Airway pressure improved to 32 mmHg with a SpO2 of 88-90%. Arterial blood gas analysis showed hypoxemia with a PO2 of 52 mmHg, PCO2 48.7, pH 7.33 and oxygen saturation of 86 per cent. FiO2 was increased to 1. Patient was haemodynamically stable throughout with a blood pressure of 110/50 mmHg, heart rate 90/min without any inotropic support. Bleeding in the ETT continued though to a lesser degree. Patient was shifted to the intensive care unit (ICU) sedated, paralyzed, on intermittent positive pressure ventilation (IPPV) with a SpO2 of 90-92 per cent, blood pressure of 102/58 mmHg, pulmonary artery pressure 29/17 mmHg and central venous pressure 8 cmH2O. Patient was ventilated with pressure regulated volume control mode with a FiO2 of 0.8 and PEEP of 8, and the peak airway pressure was 36 mmHg. Diminished chest wall movement was noticed on the left side with decreased air entry. Subsequent chest X-ray showed homogenous opacity in left lung field with mediastinal shift towards the left side suggesting left-sided lung collapse with blunting of the left costo-phrenic angle (Fig 1). Right lung field was found to be normal. Chest tubes drained a total of one-liter of blood during the first five hours. By this time, ETT bleeding had stopped. Inj noradrenaline was started at 0.05 mcg.kg^−1^.minute^−1^. Three units packed red blood cells, four units fresh frozen plasma, two units platelet concentrates and one unit platelet apheresis were transfused over the first twelve hours of post-operative period. Sixteen hours post-operatively; chest X-ray findings remained the same with progressively worsening acidosis (pH 7.22) and a PaO2 of less than 85 and base deficit of 8.9. High resolution computed tomography of the chest was done (Fig 2) which showed a clot in the left main bronchus arising from trachea (tip of ETT). In view of her clinical condition, it was decided to do a fibreoptic bronchoscopy as a diagnostic and if possible therapeutic intervention. But, fibreoptic bronchoscopy failed to dislodge and remove the clot. So the patient was prepared for emergency rigid bronchoscopy. In the operation theatre, hydrocortisone 100 mg, glycopyrrolate 0.2 mg, fentanyl 100 mcg and midazolam 1 mg was given. Anaesthesia was maintained with isoflurane, oxygen, air, fentanyl boluses and propofol infusion. After suction of the oral cavity, trachea was extubated and the rigid bronchoscope was introduced. Anaesthesia circuit was connected to the side port of the bronchoscope to maintain IPPV. The endobronchial clot was removed by forceps in piecemeal. Trachea was reintubated with 7.5 mm internal diameter ETT and IPPV was continued. Air entry improved significantly on the left side after the procedure with an increase in SpO2 to 95%. Follow-up chest radiographs revealed a well-expanded lung (Fig 3) with normal arterial blood gases. Patient was weaned from the ventilator, chest tubes were removed and trachea was extubated on the third postoperative day. She was discharged from the ICU on the fifth post-operative day without any further complications.

## Discussion

Pulmonary collapse due to an obstructing endobroncheal blood clot can complicate a variety of clinical situations. Among the potential causative factors, the most relevant to our case are pulmonary infarction^3^, pulmonary artery rupture by Swan-Ganz catheter^4^, mucosal damage resulting from suction catheter manipulation, and lung perforation or laceration as a complication of chest tube insertion^5^,^6^.

Diminished chest wall movement on the affected side, flattening of percussion note, ipsilateral tracheal deviation, decreased or absent breath sounds and reduction of the vocal fremitus characterizes the physical findings of pulmonary collapse. In ventilated patients, peak inspiratory pressure will be high with a concomitant decrease in tidal volume. Plateau pressures may or may not be elevated depending upon the degree of atelectasis. The extent of hypoxemia and hypercapnia depends on the site and degree of obstruction, associated pulmonary hemorrhage and underlying lung pathology. Interestingly, in the setting of proximal airway obstruction, the elevated airway pressure is not transmitted to the extra-parenchymal intrathoracic regions. So, venous return and cardiac output remain preserved resulting in a haemodynamically stable patient as was seen in our case, until acidosis supervenes because of the compromised respiratory function^7^. The typical roentgen appearance of pulmonary collapse is marked homogenous density on the side of the collapse, with displacement of heart, trachea and mediastinum to the affected side and elevation of the corresponding diaphragm, as was reported in our patient (Fig 1). Typical appearance of endobronchial blood clot are lobar or segmental collapse, or cut off of the air column of the trachea or main stem bronchi. The diagnosis is however confirmed by direct endoscopic evaluation of the clot.

Treatment of an endobronchial blood clot is usually supportive care until the blood clot resorbs, which usually occurs in three days^8^. However, if the patient is haemodynamically unstable or not maintaining adequate oxygen saturation, efforts at removal of the airway clot should be made urgently by way of lavage, suctioning, or forceps extraction through a flexible bronchoscope. If unsuccessful, further management options include rigid bronchoscopy, Fogarty catheter dislodgement of the clot, and topical thrombolysis with streptokinase or urokinase with partial dissolution and forceps removal^3^. For flexible fibreoptic bronchoscopy in critically ill patients during mechanical ventilation, a high FiO2, avoidance of positive end expiratory pressure, suction for short periods and frequent analysis of the arterial blood gases should be done. Anaesthetic management of rigid bronchoscopy involves adequate fasting, minimizing airway secretions, antibiotics and steroids to reduce infection and airway edema, prevention of tooth and lip injury by use of appropriate guards, control of the airway and adequate depth of general anaesthesia to limit the pressor response and suppress airway reflexes. General anaesthesia using muscle relaxants and controlled ventilation combined with topical anaesthesia of the airways in the form of local anaesthetic spray or by superior laryngeal nerve and recurrent laryngeal nerve blocks is a good option. Propofol infusion is useful in maintaining adequate depth of anaesthesia.

To conclude, endobronchial blood clot can lead to pulmonary collapse in the perioperative settings leading to haemodynamic instability and desaturation. There should be a high index of suspicion of such a clinical presentation. It need not be emphasized that timely diagnosis and management can prevent catastrophic complications.
